# *tert*-Butyl­aminium 2-carb­oxy-4,5-dichloro­benzoate

**DOI:** 10.1107/S1600536811034131

**Published:** 2011-08-27

**Authors:** Graham Smith, Urs D. Wermuth

**Affiliations:** a Faculty of Science and Technology, Queensland University of Technology, GPO Box 2434, Brisbane, Queensland 4001, Australia

## Abstract

In the structure of the title anhydrous salt, C_4_H_12_N^+^·C_8_H_3_Cl_2_O_4_^−^, the 4,5-dichloro­phthalate monoanions have the common ‘planar’ conformation with the carboxyl groups close to coplanar with the benzene ring and with a short intra­molecular carb­oxy­lic acid O—H⋯O hydrogen bond. In the crystal, a two-dimensional sheet structure is formed through aminium N—H⋯O_carbox­yl_ hydrogen-bonding associations.

## Related literature

For structures of 1:1 salts of 4,5-dichloro­phthalic acid with acyclic aliphatic amines, see: Mattes & Dorau (1986 ▶); Bozkurt *et al.* (2006 ▶); Smith & Wermuth (2010*a* ▶,*b* ▶,*c* ▶).
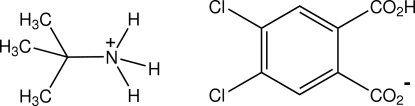


## Experimental

### 

#### Crystal data

C_4_H_12_N^+^·C_8_H_3_Cl_2_O_4_^−^
*M* *_r_* = 308.15
Monoclinic, 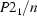

*a* = 6.1778 (2) Å
*b* = 12.7158 (4) Å
*c* = 17.7125 (7) Å
β = 96.784 (4)°
*V* = 1381.68 (8) Å^3^
*Z* = 4
Mo *K*α radiation
μ = 0.48 mm^−1^
*T* = 200 K
0.45 × 0.26 × 0.18 mm

#### Data collection

Oxford Diffraction Gemini-S CCD-detector diffractometer
Absorption correction: multi-scan (*CrysAlis PRO*; Oxford Diffraction, 2010 ▶) *T*_min_ = 0.977, *T*_max_ = 0.990
8677 measured reflections
2719 independent reflections
2307 reflections with *I* > 2σ(*I*)
*R*_int_ = 0.027

#### Refinement

*R*[*F* ^2^ > 2σ(*F* ^2^)] = 0.033
*wR*(*F* ^2^) = 0.094
*S* = 0.90
2719 reflections
188 parameters
H atoms treated by a mixture of independent and constrained refinement
Δρ_max_ = 0.25 e Å^−3^
Δρ_min_ = −0.21 e Å^−3^

### 

Data collection: *CrysAlis PRO* (Oxford Diffraction, 2010 ▶); cell refinement: *CrysAlis PRO*; data reduction: *CrysAlis PRO*; program(s) used to solve structure: *SIR92* (Altomare *et al.*, 1994 ▶); program(s) used to refine structure: *SHELXL97* (Sheldrick, 2008 ▶) within *WinGX* (Farrugia, 1999 ▶); molecular graphics: *PLATON* (Spek, 2009 ▶); software used to prepare material for publication: *PLATON*.

## Supplementary Material

Crystal structure: contains datablock(s) global, I. DOI: 10.1107/S1600536811034131/bt5620sup1.cif

Structure factors: contains datablock(s) I. DOI: 10.1107/S1600536811034131/bt5620Isup2.hkl

Supplementary material file. DOI: 10.1107/S1600536811034131/bt5620Isup3.cml

Additional supplementary materials:  crystallographic information; 3D view; checkCIF report

## Figures and Tables

**Table 1:** Hydrogen-bond geometry (Å, °)

*D*—H⋯*A*	*D*—H	H⋯*A*	*D*⋯*A*	*D*—H⋯*A*
N1*A*—H11*A*⋯O21	0.89 (2)	2.02 (2)	2.883 (2)	164 (2)
N1*A*—H12*A*⋯O11^i^	0.91 (2)	1.88 (2)	2.784 (2)	174 (2)
N1*A*—H13*A*⋯O12^ii^	0.89 (2)	1.99 (2)	2.861 (2)	167 (2)
O21—H21⋯O12	0.94 (4)	1.47 (4)	2.4021 (19)	173 (4)

Symmetry codes: (i) 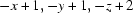; (ii) 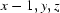.

**Fig. 1.**
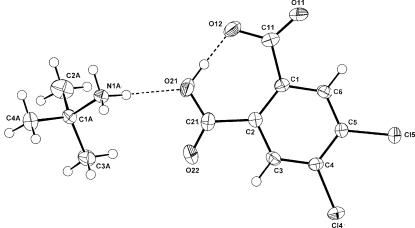
Molecular conformation and atom-numbering scheme for the title salt, with the hydrogen bonds shown as a dashed lines. Non-H atoms are shown as 50% probability displacement ellipsoids.

**Fig. 2.**
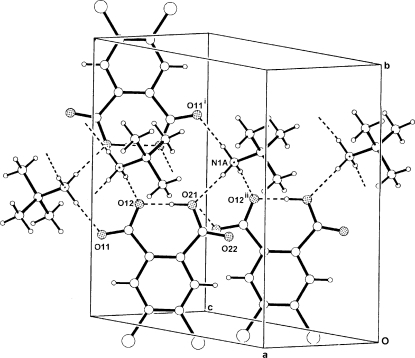
A perspective view the two-dimensional sheet structure looking down the sheet, showing hydrogen-bonding associations as dashed lines.

## supplementary crystallographic information

### Comment

4,5-Dichlorophthalic acid (DCPA) commonly forms 1:1 salts with the acyclic aliphatic amine analogues and with these, low-dimensional hydrogen-bonded structures are usually found, featuring the 'planar' hydrogen phthalate anion e.g with isopropylamine (Smith & Wermuth, 2010*a*), diisopropylamine (Smith & Wermuth, 2010*b*), diethylamine, triethylamine and *n*-butylamine (Smith & Wermuth, 2010*c*), the ammonium and tetra(*n*butyl)ammonium salts (Mattes & Dorau, 1986) and the tetramethylammonium salt (Bozkurt *et al.*, 2006). Our 1:1 stoichiometric reaction of DCPA with *t*-butylamine also gave a 1:1 salt C_4_H_12_N^+^ C_8_H_3_Cl_2_O_4_^-^, the title compound and the structure is reported here.

In this structure the common 'planar' DCPA anion is found (Fig. 1) and has the previously described (Smith & Wermuth, 2010*c*) short intramolecular carboxylic acid *O*—*H*···O_carboxy_ hydrogen bond (Table 1) (torsion angles C1–C2–C21–O22 and C2–C1–C11–O11: 176.59 (18) and 175.26 (17) Å respectively). Other structural features common to this 'planar' monoanion are a lengthening of the C1—C11 and C2—C21 bond lengths [1.522 (2) and 1.528 (3) Å] and distortion of the external bond angles at C1 and C2 [C1—C2—C21, 129.57 (15)° and C2—C1—C11, 128.84 (15)°].

Intermolecular aminium *N*—*H*···*O*(carboxyl) hydrogen bonds (Table 1) link the DCPA monoanions across *b* as well as down the *a* axis, forming a two-dimensional sheet structure (Fig. 2).

### Experimental

The title compound was synthesized by heating together for 10 min under reflux, 1 mmol quantities of 4,5-dichlorophthalic acid and *t*-butylamine in 50 ml of 50% ethanol–water. Partial evaporation of the solvent gave colourless crystalline plates from which a specimen was cleaved for the X-ray analysis..

### Refinement

Hydrogen atoms potentially involved in hydrogen-bonding interactions were located by difference methods and their positional and isotropic displacement parameters were refined. Other H atoms were included at calculated positions [C—H (aromatic) = 0.93 Å or C—H (methyl) = 0.97 Å] and treated as riding, with *U*_iso_(H) = 1.2*U*_eq_C(aromatic) or 1.5*U*_eq_*C*(methyl).

### Crystal data

**Table:**

C_4_H_12_N^+^·C_8_H_3_Cl_2_O_4_^−^	*F*(000) = 640
*M**_r_* = 308.15	*D*_x_ = 1.481 Mg m^−^^3^
Monoclinic, *P*2_1_/*n*	Mo *K*α radiation, λ = 0.71073 Å
Hall symbol: -P 2yn	Cell parameters from 4272 reflections
*a* = 6.1778 (2) Å	θ = 3.3–28.8°
*b* = 12.7158 (4) Å	µ = 0.48 mm^−^^1^
*c* = 17.7125 (7) Å	*T* = 200 K
β = 96.784 (4)°	Plate, colourless
*V* = 1381.68 (8) Å^3^	0.45 × 0.26 × 0.18 mm
*Z* = 4	

### Data collection

**Table:**

Oxford Diffraction Gemini-S CCD-detector diffractometer	2719 independent reflections
Radiation source: Enhance (Mo) X-ray source	2307 reflections with *I* > 2σ(*I*)
graphite	*R*_int_ = 0.027
Detector resolution: 16.077 pixels mm^-1^	θ_max_ = 26.0°, θ_min_ = 3.4°
ω scans	*h* = −7→7
Absorption correction: multi-scan (*CrysAlis PRO*; Oxford Diffraction, 2010)	*k* = −15→15
*T*_min_ = 0.977, *T*_max_ = 0.990	*l* = −21→21
8677 measured reflections	

### Refinement

**Table:**

Refinement on *F*^2^	Primary atom site location: structure-invariant direct methods
Least-squares matrix: full	Secondary atom site location: difference Fourier map
*R*[*F*^2^ > 2σ(*F*^2^)] = 0.033	Hydrogen site location: inferred from neighbouring sites
*wR*(*F*^2^) = 0.094	H atoms treated by a mixture of independent and constrained refinement
*S* = 0.90	*w* = 1/[σ^2^(*F*_o_^2^) + (0.0608*P*)^2^ + 0.4558*P*] where *P* = (*F*_o_^2^ + 2*F*_c_^2^)/3
2719 reflections	(Δ/σ)_max_ = 0.001
188 parameters	Δρ_max_ = 0.25 e Å^−^^3^
0 restraints	Δρ_min_ = −0.21 e Å^−^^3^

### Special details

**Table:**

Geometry. Bond distances, angles *etc*. have been calculated using the rounded fractional coordinates. All su's are estimated from the variances of the (full) variance-covariance matrix. The cell e.s.d.'s are taken into account in the estimation of distances, angles and torsion angles
Refinement. Refinement of *F*^2^ against ALL reflections. The weighted *R*-factor *wR* and goodness of fit *S* are based on *F*^2^, conventional *R*-factors *R* are based on *F*, with *F* set to zero for negative *F*^2^. The threshold expression of *F*^2^ > σ(*F*^2^) is used only for calculating *R*-factors(gt) *etc*. and is not relevant to the choice of reflections for refinement. *R*-factors based on *F*^2^ are statistically about twice as large as those based on *F*, and *R*- factors based on ALL data will be even larger.

### Fractional atomic coordinates and isotropic or equivalent isotropic displacement parameters (Å^2^)

**Table:**

	*x*	*y*	*z*	*U*_iso_*/*U*_eq_	
Cl1	0.77566 (7)	−0.08494 (3)	0.92701 (3)	0.0313 (1)	
Cl2	0.29591 (7)	−0.09103 (3)	0.84148 (3)	0.0370 (2)	
O11	0.8896 (2)	0.29012 (10)	1.01297 (8)	0.0394 (4)	
O12	0.6289 (2)	0.40012 (9)	0.97033 (8)	0.0353 (4)	
O21	0.2615 (2)	0.39679 (10)	0.90835 (10)	0.0452 (5)	
O22	0.0363 (2)	0.28642 (11)	0.84456 (9)	0.0475 (5)	
C1	0.5836 (3)	0.21454 (12)	0.93969 (9)	0.0218 (5)	
C2	0.3705 (3)	0.21275 (13)	0.90024 (9)	0.0230 (5)	
C3	0.2876 (3)	0.11656 (13)	0.87218 (10)	0.0239 (5)	
C4	0.4066 (3)	0.02459 (12)	0.87963 (9)	0.0233 (5)	
C5	0.6169 (3)	0.02693 (12)	0.91703 (9)	0.0221 (5)	
C6	0.7012 (3)	0.12081 (13)	0.94635 (9)	0.0236 (5)	
C11	0.7102 (3)	0.30722 (13)	0.97701 (9)	0.0268 (5)	
C21	0.2089 (3)	0.30285 (14)	0.88234 (11)	0.0311 (6)	
N1A	−0.0938 (3)	0.54459 (13)	0.90362 (9)	0.0257 (5)	
C1A	−0.2152 (3)	0.58740 (13)	0.83097 (10)	0.0255 (5)	
C2A	−0.0614 (3)	0.66054 (18)	0.79557 (12)	0.0450 (7)	
C3A	−0.2819 (3)	0.49408 (16)	0.77986 (11)	0.0380 (6)	
C4A	−0.4120 (3)	0.64663 (15)	0.85216 (12)	0.0373 (6)	
H3	0.14630	0.11430	0.84740	0.0290*	
H6	0.84200	0.12160	0.97160	0.0280*	
H21	0.400 (6)	0.399 (3)	0.936 (2)	0.109 (12)*	
H11A	0.016 (3)	0.5023 (18)	0.8953 (12)	0.039 (6)*	
H12A	−0.036 (4)	0.5995 (18)	0.9325 (13)	0.044 (6)*	
H13A	−0.185 (4)	0.5080 (18)	0.9289 (13)	0.042 (6)*	
H21A	0.06120	0.62120	0.78220	0.0670*	
H22A	−0.01140	0.71450	0.83140	0.0670*	
H23A	−0.13660	0.69220	0.75070	0.0670*	
H31A	−0.37940	0.44980	0.80390	0.0570*	
H32A	−0.15460	0.45460	0.77120	0.0570*	
H33A	−0.35380	0.51880	0.73220	0.0570*	
H41A	−0.50720	0.59880	0.87410	0.0560*	
H42A	−0.48830	0.67810	0.80740	0.0560*	
H43A	−0.36510	0.70060	0.88840	0.0560*	

### Atomic displacement parameters (Å^2^)

**Table:**

	*U*^11^	*U*^22^	*U*^33^	*U*^12^	*U*^13^	*U*^23^
Cl1	0.0293 (2)	0.0232 (2)	0.0394 (3)	0.0066 (2)	−0.0041 (2)	−0.0007 (2)
Cl2	0.0298 (3)	0.0252 (2)	0.0534 (3)	−0.0039 (2)	−0.0061 (2)	−0.0113 (2)
O11	0.0394 (8)	0.0314 (7)	0.0433 (8)	−0.0070 (6)	−0.0116 (6)	−0.0082 (6)
O12	0.0367 (7)	0.0208 (6)	0.0486 (8)	−0.0044 (5)	0.0059 (6)	−0.0061 (5)
O21	0.0321 (8)	0.0228 (7)	0.0798 (11)	0.0057 (6)	0.0025 (8)	−0.0041 (7)
O22	0.0377 (8)	0.0409 (8)	0.0591 (10)	0.0146 (6)	−0.0148 (7)	−0.0028 (7)
C1	0.0250 (8)	0.0205 (8)	0.0198 (8)	−0.0024 (6)	0.0028 (7)	−0.0010 (6)
C2	0.0228 (8)	0.0233 (8)	0.0231 (8)	0.0017 (6)	0.0031 (7)	0.0010 (6)
C3	0.0180 (8)	0.0274 (8)	0.0257 (8)	−0.0004 (7)	0.0004 (7)	−0.0007 (7)
C4	0.0231 (8)	0.0216 (8)	0.0249 (8)	−0.0033 (6)	0.0012 (7)	−0.0026 (7)
C5	0.0223 (8)	0.0213 (8)	0.0225 (8)	0.0018 (6)	0.0018 (6)	0.0016 (6)
C6	0.0204 (8)	0.0265 (8)	0.0228 (8)	−0.0016 (6)	−0.0020 (7)	−0.0007 (7)
C11	0.0311 (9)	0.0247 (9)	0.0251 (9)	−0.0068 (7)	0.0049 (8)	−0.0032 (7)
C21	0.0316 (10)	0.0258 (9)	0.0361 (10)	0.0067 (7)	0.0053 (8)	0.0027 (8)
N1A	0.0236 (8)	0.0251 (8)	0.0268 (8)	0.0001 (7)	−0.0037 (7)	−0.0033 (6)
C1A	0.0245 (9)	0.0262 (9)	0.0246 (9)	0.0016 (7)	−0.0021 (7)	−0.0001 (7)
C2A	0.0437 (12)	0.0542 (13)	0.0374 (11)	−0.0117 (10)	0.0066 (9)	0.0081 (10)
C3A	0.0385 (11)	0.0404 (11)	0.0315 (10)	0.0050 (9)	−0.0108 (8)	−0.0097 (8)
C4A	0.0328 (10)	0.0329 (10)	0.0451 (11)	0.0079 (8)	0.0004 (9)	0.0001 (9)

### Geometric parameters (Å, °)

**Table:**

Cl1—C5	1.7251 (17)	C4—C5	1.387 (3)
Cl2—C4	1.7253 (16)	C5—C6	1.379 (2)
O11—C11	1.231 (2)	C3—H3	0.9300
O12—C11	1.284 (2)	C6—H6	0.9300
O21—C21	1.307 (2)	C1A—C2A	1.517 (3)
O22—C21	1.208 (2)	C1A—C3A	1.520 (3)
O21—H21	0.94 (4)	C1A—C4A	1.515 (3)
N1A—C1A	1.512 (2)	C2A—H21A	0.9600
N1A—H11A	0.89 (2)	C2A—H22A	0.9600
N1A—H13A	0.89 (2)	C2A—H23A	0.9600
N1A—H12A	0.91 (2)	C3A—H31A	0.9600
C1—C11	1.522 (2)	C3A—H32A	0.9600
C1—C6	1.394 (2)	C3A—H33A	0.9600
C1—C2	1.416 (3)	C4A—H41A	0.9600
C2—C3	1.395 (2)	C4A—H42A	0.9600
C2—C21	1.528 (3)	C4A—H43A	0.9600
C3—C4	1.379 (2)		
			
Cl1···Cl2	3.1661 (7)	O11···C21^v^	3.217 (2)
Cl1···O11^i^	3.4197 (14)	O11···Cl1^i^	3.4197 (14)
Cl1···C3^ii^	3.6461 (18)	O11···N1A^vi^	2.784 (2)
Cl2···Cl1	3.1661 (7)	O12···C21	3.120 (2)
Cl1···H43A^iii^	2.9200	O12···O12^vi^	3.2387 (17)
Cl1···H6^i^	2.8300	O12···O21	2.4021 (19)
Cl2···H22A^iv^	3.1100	O12···N1A^v^	2.861 (2)
O11···O22^v^	3.219 (2)	O21···C11	3.109 (2)
			
C21—O21—H21	113 (2)	C4—C3—H3	119.00
C1A—N1A—H11A	112.8 (14)	C1—C6—H6	119.00
C1A—N1A—H12A	108.9 (14)	C5—C6—H6	119.00
H11A—N1A—H12A	107 (2)	C3A—C1A—C4A	111.52 (15)
H11A—N1A—H13A	108 (2)	N1A—C1A—C2A	107.49 (15)
C1A—N1A—H13A	109.6 (15)	N1A—C1A—C3A	107.35 (14)
H12A—N1A—H13A	110 (2)	N1A—C1A—C4A	107.46 (15)
C2—C1—C11	128.84 (15)	C2A—C1A—C3A	111.81 (16)
C6—C1—C11	112.93 (15)	C2A—C1A—C4A	110.97 (15)
C2—C1—C6	118.24 (15)	C1A—C2A—H21A	109.00
C1—C2—C21	129.57 (15)	C1A—C2A—H22A	109.00
C1—C2—C3	118.09 (15)	C1A—C2A—H23A	109.00
C3—C2—C21	112.35 (16)	H21A—C2A—H22A	109.00
C2—C3—C4	122.71 (17)	H21A—C2A—H23A	109.00
Cl2—C4—C5	120.72 (12)	H22A—C2A—H23A	109.00
Cl2—C4—C3	120.19 (14)	C1A—C3A—H31A	110.00
C3—C4—C5	119.08 (15)	C1A—C3A—H32A	109.00
Cl1—C5—C4	121.35 (12)	C1A—C3A—H33A	109.00
Cl1—C5—C6	119.36 (14)	H31A—C3A—H32A	109.00
C4—C5—C6	119.28 (15)	H31A—C3A—H33A	109.00
C1—C6—C5	122.58 (17)	H32A—C3A—H33A	109.00
O11—C11—C1	118.25 (15)	C1A—C4A—H41A	109.00
O11—C11—O12	121.94 (16)	C1A—C4A—H42A	109.00
O12—C11—C1	119.82 (15)	C1A—C4A—H43A	109.00
O21—C21—C2	118.87 (16)	H41A—C4A—H42A	110.00
O21—C21—O22	121.30 (17)	H41A—C4A—H43A	109.00
O22—C21—C2	119.83 (16)	H42A—C4A—H43A	109.00
C2—C3—H3	119.00		
			
C6—C1—C2—C3	1.9 (2)	C1—C2—C21—O21	−3.2 (3)
C6—C1—C2—C21	−177.99 (17)	C1—C2—C21—O22	176.59 (18)
C11—C1—C2—C3	−178.68 (16)	C3—C2—C21—O21	176.90 (17)
C11—C1—C2—C21	1.4 (3)	C3—C2—C21—O22	−3.3 (2)
C2—C1—C6—C5	−0.9 (2)	C2—C3—C4—Cl2	−178.43 (14)
C11—C1—C6—C5	179.57 (15)	C2—C3—C4—C5	0.5 (3)
C2—C1—C11—O11	175.26 (17)	Cl2—C4—C5—Cl1	−0.1 (2)
C2—C1—C11—O12	−4.8 (3)	Cl2—C4—C5—C6	179.48 (13)
C6—C1—C11—O11	−5.3 (2)	C3—C4—C5—Cl1	−179.02 (13)
C6—C1—C11—O12	174.65 (15)	C3—C4—C5—C6	0.5 (2)
C1—C2—C3—C4	−1.8 (3)	Cl1—C5—C6—C1	179.23 (13)
C21—C2—C3—C4	178.14 (16)	C4—C5—C6—C1	−0.3 (3)

Symmetry codes: (i) −*x*+2, −*y*, −*z*+2; (ii) −*x*+1, −*y*, −*z*+2; (iii) *x*+1, *y*−1, *z*; (iv) *x*, *y*−1, *z*; (v) *x*+1, *y*, *z*; (vi) −*x*+1, −*y*+1, −*z*+2.

### Hydrogen-bond geometry (Å, °)

**Table:**

*D*—H···*A*	*D*—H	H···*A*	*D*···*A*	*D*—H···*A*
N1A—H11A···O21	0.89 (2)	2.02 (2)	2.883 (2)	164 (2)
N1A—H12A···O11^vi^	0.91 (2)	1.88 (2)	2.784 (2)	174 (2)
N1A—H13A···O12^vii^	0.89 (2)	1.99 (2)	2.861 (2)	167 (2)
O21—H21···O12	0.94 (4)	1.47 (4)	2.4021 (19)	173 (4)
C3—H3···O22	0.93	2.29	2.671 (2)	104
C6—H6···O11	0.93	2.27	2.657 (2)	104

Symmetry codes: (vi) −*x*+1, −*y*+1, −*z*+2; (vii) *x*−1, *y*, *z*.
